# Lesion Volume in a Bi- or Multivariate Prediction Model for the Management of PI-RADS v2.1 Score 3 Category Lesions

**DOI:** 10.5152/tud.2022.22038

**Published:** 2022-07-01

**Authors:** Eugenio Martorana, Maria Cristina Aisa, Riccardo Grisanti, Nicola Santini, Giacomo Maria Pirola, Alessandro Datti, Sandro Gerli, Alessandra Bonora, Aldo Burani, Giovanni Battista Scalera, Pietro Scialpi, Aldo Di Blasi, Michele Scialpi

**Affiliations:** 1 Department of Urology, New Civilian Hospital of Sassuolo, Modena, Italy; 2 Division of Gynaecology, Department of Medicine and Surgery, S. Maria della Misericordia Hospital, Perugia University, Perugia, Italy; 3 Department of Radiology, New Civilian Hospital of Sassuolo, Modena, Italy; 4 Department of Urology, Usl Toscana Sud Est, San Donato Hospital, Arezzo, Italy; 5 Division of Biochemistry, Department of Agricultural, Food, and Environmental Sciences, Perugia University, Perugia, Italy; 6 Division of Anaesthesia, Casa di Cura Fogliani, Modena, Italy; 7 Division of Diagnostic Imaging, Department of Medicine and Surgery, S. Maria della Misericordia Hospital, Perugia University, Italy; 8 Division of Urology, Portogruaro Hospital, Venice, Italy; 9 Division of Radiology, Tivoli Hospital, Tivoli, Italy; 10 Division of Radiology 2, Department of Medicine and Surgery, S. Maria della Misericordia Hospital, Perugia University, Perugia, Italy

**Keywords:** Lesion volume, 0.5 mL cutoff, transperineal MRI/TRUS fusion-targeted biopsy

## Abstract

**Objective:**

This study aimed at improving the discrimination of Prostate Imaging – Reporting and Data System version 2.1 (PI-RADS v2.1) score 3 suspicious prostate cancer lesions using lesion volume evaluation.

**Material and methods:**

Two hundred five PI-RADS v2.1 score 3 lesions were submitted to transperineal MRI/TRUS fusion-targeted biopsy. The lesion volumes were estimated on diffusion-weighted imaging sequence and distributed in PI-RADS 3a (LV < 0.5 mL) and PI-RADS 3b (LV ≥ 0.5 mL) subcategories, using a 0.5 mL cutoff value. Data were retrospectively matched with histopathological findings from the biopsy. Assuming that lesions with LV < or ≥ 0.5 mL were respectively not eligible (benign and indolent PCa lesions) or eligible for biopsy (significant PCa lesions), the diagnostic accuracy of lesion volume in determining clinically significant PCa at biopsy was evaluated using a bi- or multivariate model.

**Results:**

About 55.1% and 44.9% of lesions were distributed in subcategories 3a and 3b, respectively. The overall PI-RADS score 3 detection rate was 273%. 3.5% (1.95% of total), and 25% (11.7% of total) significant PCa were found in PI-RADS 3a and 3b subcategory, respectively. The method showed 85.2% sensitivity, 61.2% specificity, 25% positive predictive value, and 96.5% negative predictive value and avoided 55.1% of unnecessary biopsies. The diagnostic accuracy in determining significant PCa at biopsy was 73.2% or 86.5% depending on whether lesion volume was used alone or in combination with prostate volume and patient age in a multivariate model.

**Conclusion:**

0.5 mL lesion volume cutoff value significantly discriminates fusion-targeted biopsy need in PI-RADS v2.1 score 3 lesions and its diagnostic accuracy improves when it combines with prostate volume and age in a multivariate model.

**Main Points**

- The presence of clinically significant prostate cancer in PI-RADS score 3 category is equivocal, therefore, in clinical practice, a definitive managing consensus (i.e., target biopsy or, alternatively, clinical surveillance) for PI-RADS score 3 or equivocal lesions is lacking.
- Assuming that PI-RADS score 3 lesions with volume < and ≥ 0.5 mL were respectively not eligible (benign/indolent PCa) or eligible (significant PCa) for biopsy, this categorization approach showed a 73.2% diagnostic accuracy in determining the presence of clinically significant PCa at biopsy avoiding 55.1% of unnecessary biopsies.
- Combining lesion volume with prostate volume and patient age in a multivariate model, the diagnostic accuracy increased to 86.5%.
- Lesion volume detection and categorization, using 0.5 mL cutoff value, allows for a significant recognition of PI-RADS v2.1 score 3 lesions to be biopsied.

## Introduction

Currently, multiparametric magnetic resonance imaging (mpMRI) has a fundamental role in diagnosis, treatment monitoring, and outcome prediction for prostate cancer (PCa) management.

Although it has been widely demonstrated that PI-RADS v2.1 has significantly improved both the detection and localization of PCa, providing a clinical guideline with the assessment of the 5 risk categories for each examination,^1^ it shows some potential ambiguities and gaps^2^ which need to be overcome. In particular, PI-RADS v2.1 is a risk assessment system for PCa based on MRI characteristics. Nevertheless, clear guidance for clinical management should actually be carried out including patient history (i.e., age, number of previous biopsies, and diagnosis at previous biopsies), biochemical characteristics (i.e., PSA level, PSA velocity, and PSA density), familiar factors and not only MRI appearance. Moreover, a major limitation of PI-RADS v2.1 score is represented by PI-RADS score 3 lesion category which is defined as equivocal for the presence of clinically significant PCa (sPCa). In clinical practice, this results in the lack of a definitive managing consensus (i.e., target biopsy or, alternatively, clinical surveillance). Also, the detection of PCa within PI-RADS 3 lesions depends on many factors. These include the experience of the biopsy surgeon and the experience of the radiologist in interpreting the MRI images. Several studies have demonstrated that the PCa detection rate in biopsied PI-RADS score 3 lesions has significant high variability, ranging from 5% to 26% (including a low rate for significant cancer).^3,4^ This percentage increases significantly considering the experienced centers that reported a PCa detection rate up to 44% with a sPCa detection of 34%.^5^

Thus, the identification of a reliable discriminating factor within PI-RADS v2.1 score 3 category should be a key point in identifying appropriate patient-tailored management options (which may include targeted biopsy versus clinical surveillance) in order to increase the diagnostic accuracy of sPCa and to reduce the overdetection of iPCa.

A major diagnostic potential of mpMRI lesion volume (LV) for PCa and tumor aggressiveness has been reported and a higher detection of sPCa for LVs ≥ 0.5 mL has been evidenced.^6^

In the present study, looking for improved decision-making in PI-RADS v2.1 score 3 lesion category, we evaluated the diagnostic accuracy of LV classification approach in detecting sPCa at biopsy using a cutoff value of 0.5 mL. This assessment was also done in combination with other factors, including age, PSA, prostate volume (PV), and PSA density (PSAD) in a multivariate model.

## Materials and Methods

### Inclusion Criteria of Patients and Characteristics of the Lesion Database Considered for the Study

The indication for mpMRI examination was applied to all patients with (a) persistently high serum PSA level; (b) previous diagnosis of atypical small acinar proliferation (ASAP) or multifocal high-grade prostatic intraepithelial neoplasia (HGPIN); and/or (c) suspicious digital rectal examination.

The database considered for the study was from a single-center casuistry and included 205 PI-RADS v2.1 score 3 lesions that were submitted to transperineal MRI/TRUS fusion-targeted biopsy (FTBx) using the Esaote MyLab 9 ® system (Esaote, Genoa, Italy).

The Institutional Review Board approved the present retrospective investigation. As data were provided in an anonymous format, the Institutional Review Board waived the requirement to obtain informed consent and ethical committee approval was received from Comitato Etico Regionale (CER) Umbria, Italia (CER 4338/22). The study was compliant with the Health Insurance Portability and Accountability Act.

### Evaluated Outcomes

The outcomes considered in this study were assessed as follows

- (a) Any PCa detection to determine the overall cancer detection rate in PI-RADS v2.1 score 3 lesion category.
- (b) Epstein’s criteria^7^ (i.e., Gleason score ≥ 3 + 4, or any Gleason score with tumor volume ≥ 0.5 mL, or extra-prostatic extension) to define sPCa detection rate.
- (c) Correspondence between MRI LV and histological tumor volume^6,8,9^ to evaluate cancer volume if PCa was detected after target biopsy.

### Prostatic mpMRI Parameters

All mpMRI in this study was performed using a 1.5 Tesla (Ingenia, Philips) and a 3.0 Tesla (Achieva, Philips) with a superficial body phased array coil with 32 and 16 channels, respectively.

### The protocol for prostate MRI included

- axial, sagittal, and coronal T2-weighted (T2W) turbo-spin-echo (TSE) imaging; T2W axial image slice thickness was 3 mm with no gap and acquisition resolution of 0.44 × 0.44 mm;
- axial diffusion-weighted imaging (DWI; b values of b 50, 500, 1000 s/mm^2^ + single b value of 1600) with apparent diffusion coefficient (ADC) map reconstructions;
- axial T1-weighted Gradient echo fat suppression dynamic contrast-enhanced (DCE) MRI; DCE temporal resolution was 10 s for 2 : 33 min (15 phases) without breath holding, ­following an intravenous single dose of 0.2 mL/kg at 2.0 mL/s of contrast Gadoteric Acid 0.5 mmol/mL (Dotarem, Guerbet); and
- axial T1-weighted Dixon 3D after contrast with fat suppression.

The axial images were orientated on the same plane referred to the urethra line. Only qualitative analysis for DWI and DCE MRI was carried out.

**Figure 1. a-d.**
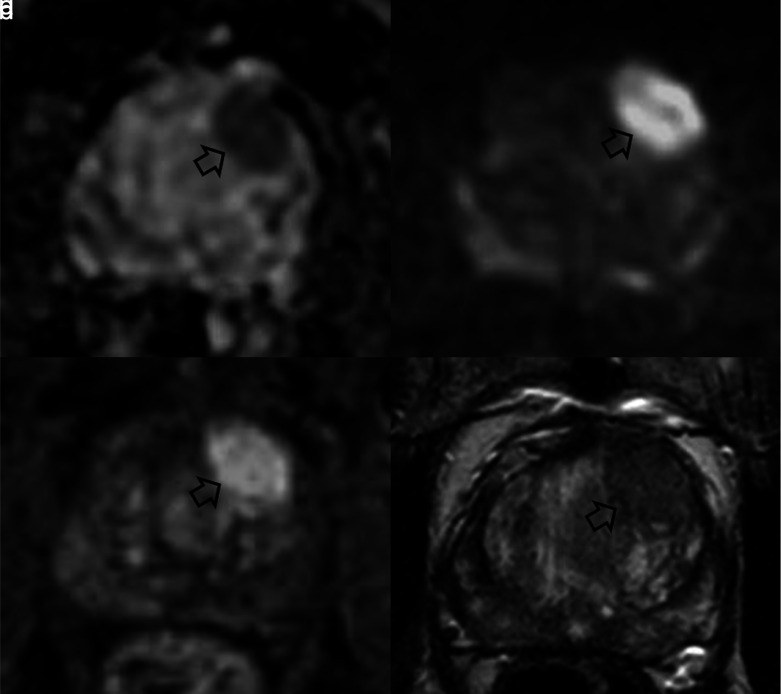
Multiparametric MRI in a 63-year-old man with persistently elevated serum PSA level (8.4 ng/mL). PIRADS score 3b lesion in the left anterior transition zone to the middle of the prostate gland. Target biopsy was performed. The lesion is moderately hypo-intense on the ADC map (arrow in a), hyper-intense on DWI at high b-values (arrow in b) with early intense enhancement on DCE (arrow in c), and moderately hypointense on the T2-weighted image (arrow in d). GS 7 (3+4) prostate adenocarcinoma in 4 out of 4 target cores was found at histology. No cancer was detected in the peripheral zone at the random biopsy (8 cores). The patient underwent radical prostatectomy and a final histopathological examination confirmed the biopsy findings (prostate adenocarcinoma GS 3+4 pT2c, R0, N0, Mx).

Two radiologists, with a good experience in prostate MRI (N.S. and M.S.), evaluated the mpMRI, searched for the presence of any PI-RADS v2.1 score 3 suspicious lesions, and reached a consensus. Finally, the location of the PI-RADS v2.1 score 3 lesions was recorded according to the 38 PI-RADS v2.1 prostatic sectors (**Figure 1**).

### LV Calculation

Radiological LV was determined using the ellipsoid formula (i.e., assial × sagittal × coronal diameter × 0.523) on DWI sequences. The calculated volumes, expressed in milliliter (mL), were recorded in our database for each PI-RADS v2.1 score 3 lesion. A slight inter-observational variability was detected in 4% of the cases and related disagreement was solved reaching a consensus among the radiologists. No events of classification of the same lesion within different PI-RADS score categories (i.e., 2, 3, or 4), indicated by the readers, occurred.

### Biopsy Technique

An evacuative enema was required to clean the rectal ampoule 12 and 3 hours before the biopsy.

Each procedure was preceded by a patient interview in which the risks/benefits of the biopsy were examined, and the biopsy technique was explained again to the patient through explanatory material, RM images, and drawings. Informed consent was signed for each procedure. During the phase of the Covid-19 emergency, patients were also informed about the extraordinary procedures for the prevention of infections, by having a specific consent signed.

Fusion biopsy was carried out in an outpatient setting in a dedicated surgery room with a short observation space. All the patients were placed in a lithotomy position by opening the lower limbs in order to obtain the widest window possible at the level of the perineum. The antibiotic prophylaxis was administered 30 minutes before, intravenously (amoxicillin-clavulanic acid 2.2 g/L). Before insertion of the biplanar-endorectal ultrasound probe (TLC 3-13, Esaote) digital-rectal exploration and adequate preparation of the field were carried out. The procedure was performed under ultrasound-guided local anesthesia with 2% lidocaine on the prostatic apex through single access approximately 1.5 cm above the anal orifice.

For the ECO/MRI images fusion, the axial and sagittal T2 weight sequences and the axial ADC map were imported into the ultrasound device (Esaote®, MyLab 9, Genoa, Italy), aligning them and demarking/turning each suspicious area using a specific application system (Virtual Navigator Urofusion, Esaote spa). Real-time fusion was achieved through continuous communication between the ultrasound probe equipped with a tracking device and a magnet, which was placed near the patient and continuously was verifying spatial coordinates of suspicious areas inside the prostate.

**Figure 2. a-d.**
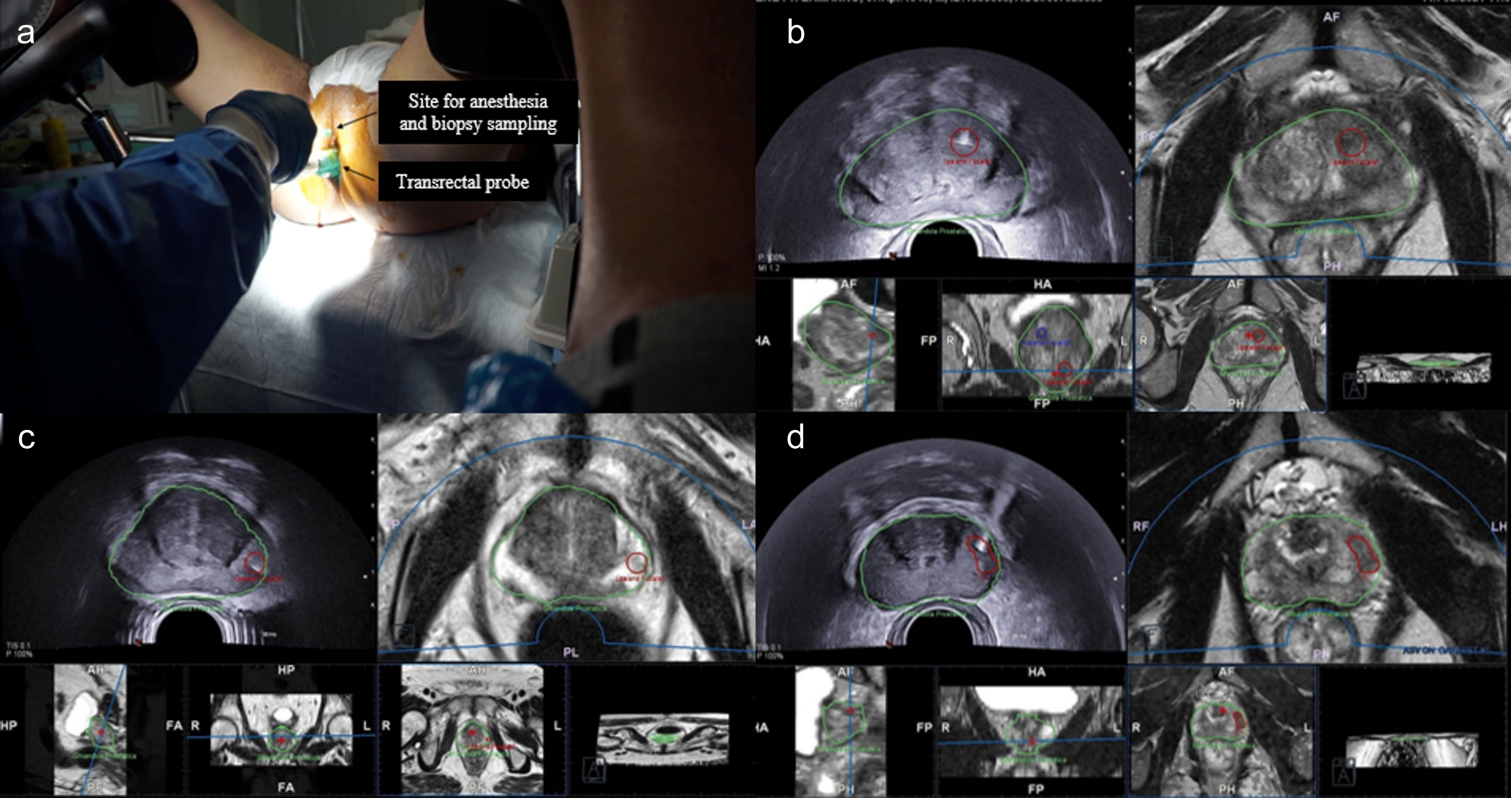
Target biopsy. The access to the gland was carried out using a single access transperineal approach with local anesthesia (a). A PIRADS 3a score lesion was biopsied respectively in the transition zone (b) and in the peripheral zone (c). A PIRADS 3b score was assigned in the last case (d). Histopathological findings detected an HGPIN, and a GS 6 (3+3) prostate adenocarcinoma in 2/4 target cores and a GS 6 (3+3) prostate adenocarcinoma in 4 out of 4 target cores respectively on b, c, and d examinations. Active Surveillance and curative treatment (radical prostatectomy) were carried out for c and d cases, respectively.

The accuracy of image fusion was evaluated by sliding the probe from apex to basis of the gland or vice versa. Thus, the targeted biopsy was carried out using an 18 G needle by making a minimum of 3 to a maximum of 5 samples for each lesion and accessing the gland through the same via used for anesthesia (**Figure 2**). Random samples were then performed on peripheral prostate parenchyma with a maximum of 14 samples (including target samples). A compression dressing was performed on the needle at the access point at the end of the procedure. The patient was kept under observation until urination and was discharged with the appropriate recommendations.

The present approach represents an advancement of the cognitive fusion one. Compared to this, it indeed entails the advantage of software assisting the fusion process which eliminates the cognitive effort of the operator. In addition, it implicates the “freehand” mode biopsy technique which avoids possible conflicts between biopsy needle and pelvic skeleton, especially for the sampling of anterior areas in very large prostates that represents the major limits of template guided procedures.^10^

### Statistical Analysis

Data analysis and graphs were carried out using IBM SPSS Statistics v.23 (IBM SPSS Corp.; Armonk, NY, USA) and GraphPad Prism (version 6.01) statistical software, respectively.

D’Agostino and Pearson’s normality test was preliminarily used to assess the normal distribution of variables. Using 0.5 mL LV cutoff value to distinguish lesions to be biopsied (biopsy for LV ≥ cutoff) in PI-RADS 3 category, the diagnostic accuracy of this procedure was evaluated assessing different parameters (i.e., Sensitivity; Specificity; Positive Predictive Value, PPV; Negative Predictive Value, NPV; Overall Diagnostic Accuracy).

Results of age, LV, prostate volume (PV), PSA and PSA density (PSAD) were presented in median and interquartile ranges. As data were not normally distributed, comparison between the two groups was performed using the non-parametric Mann–Whitney test. The diagnostic accuracy of LV and the best cutoff value was evaluated by measuring the area under the ROC curve (AUC) that was performed by comparing benign/iPCa lesions versus the clinically significant ones. This univariable accuracy analysis was also performed for the other variables measured in our study, including LV categorized (LVencod) in two levels (0 for LV <0.5 mL and 1 for LV ≥0.5 mL). Bivariate and multivariate analysis approach to test the ability of all predictors in determining the presence of clinically sPCa at biopsy in PI-RADS 3 category was done using the binary logistic regression. The respective odds ratio (OR) with 95% CIs were calculated. The logistic regression model incorporated as explanatory variables all variables that showed a corrected *P*-value (pc) ≤ 0.25 in bivariate analysis. To avoid multicollinearity problems, predictors in strong correlation with other explanatory variables were dropped from the model. Logistic regression was then complemented by predictive accuracy test that was quantified as the AUC.

## Results

### Characteristics of Lesions in PI-RADS v2.1 Score 3 Category with Reference to mpMRI and Histological Analysis

According to MRI LV estimation, PI-RADS V2.1 score 3 lesions were distinguished into two groups: (1) PI-RADS 3a which included lesions with volume < 0.5 mL and (2) PI-RADS 3b which included lesions with volume ≥ 0.5 mL.

Results of MRI and histopathological analysis of PI-RADS v2.1 score 3 category and subcategories (PI-RADS 3a and PI-RADS 3b) are resumed in Table 1.

Of the 205 investigated lesions, 113 (55.1%) were classified as PI-RADS 3a and 92 (44.9%) as PI-RADS 3b. The overall PCa detection rate was 27.3% (56/205 lesions).

In PI-RADS 3a lesions, 109 lesions (96.5%) included 80 benign lesions (73.4%) and 29 iPCa (26.6%). By contrast, 4 lesions (3.5%) were diagnosed as sPCa (corresponding to 1.95% of entire PI-RADS V2.1 score 3 risk category).

In PI-RADS 3b lesions, 69 lesions (75%) were diagnosed as benign disease whereas 23 lesions (25%) were diagnosed as sPCa (corresponding to 11.7% of the entire score 3 risk category).

### Diagnostic Accuracy of the LV Classification Approach Using 0.5 mL Cutoff Value

Assuming that LVs with values < and ≥ to 0.5 mL were respectively not eligible (benign/iPCa lesions) or eligible (sPCa lesions) for biopsy and consequently were test negatives or test positives, the diagnostic accuracy of the LV classification approach in determining the presence of clinically sPCa at biopsy was retrospectively evaluated according to the histopathological data. Results indicated a sensitivity and specificity rate of 85.2% and 61.2%, respectively. The positive predictive value (PPV) which corresponded to the overall detection rate of sPCa was 25%. The negative predictive value (NPV) was 96.5% to which 3.5%, corresponding to ~1.95% of the total, of undetected sPCa lesions was related.

### Variability of PSA, PV, PSAD, and LV in PI-RADS 3 Category and Subcategories or in the Groups of Benign/iPCa and sPCa Lesions

Variability (expressed as a median and interquartile range) of LV, age, PSA, PV and PSAD levels were measured in PI-RADS 3 category and subcategories (Table 2) or in the groups of benign/iPCa and sPCa lesions (Table 3).

**Figure 3.**
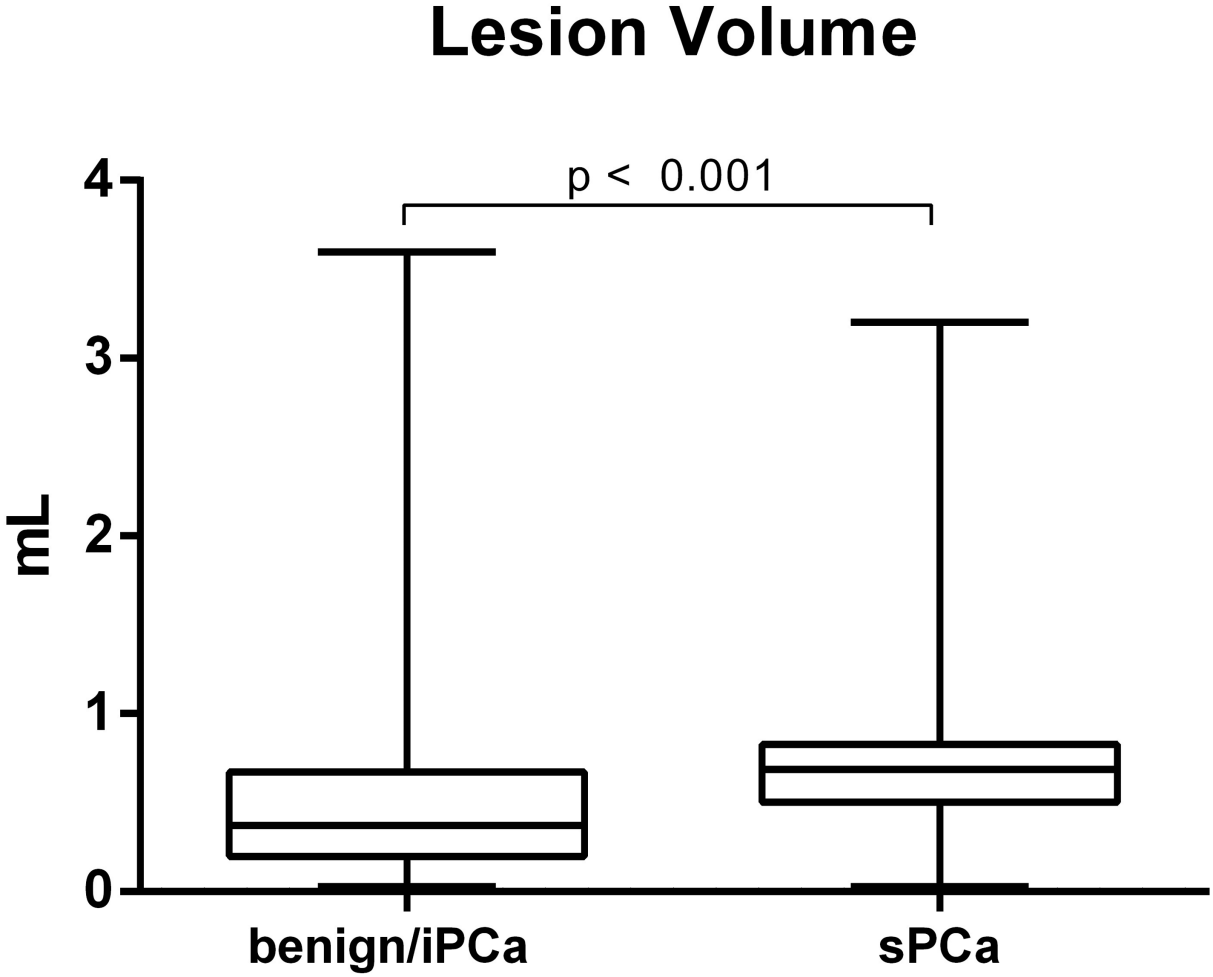
Lesion volume in benign/indolent and significant PCa lesions.

Statistical comparison of variables between PI-RADS 3 subcategories or between benign/iPCa and sPCa lesions was also performed and results are reported in Tables 2 and 3, respectively. In detail, LV and PV demonstrated a significant increase in PI-RADS 3b compared to PI-RADS 3a, whereas PSAD was statistically reduced. On the other hand, sPCa lesions versus benign/iPCa ones showed significantly higher values of LV and significantly lower values of PV. Figure 3 graphically represents the variability of LV in the groups of benign/iPCa and sPCa lesions.

### ROC Curve of LV, LVencod, Age, PSA, PV, and PSAD

**Figure 4.**
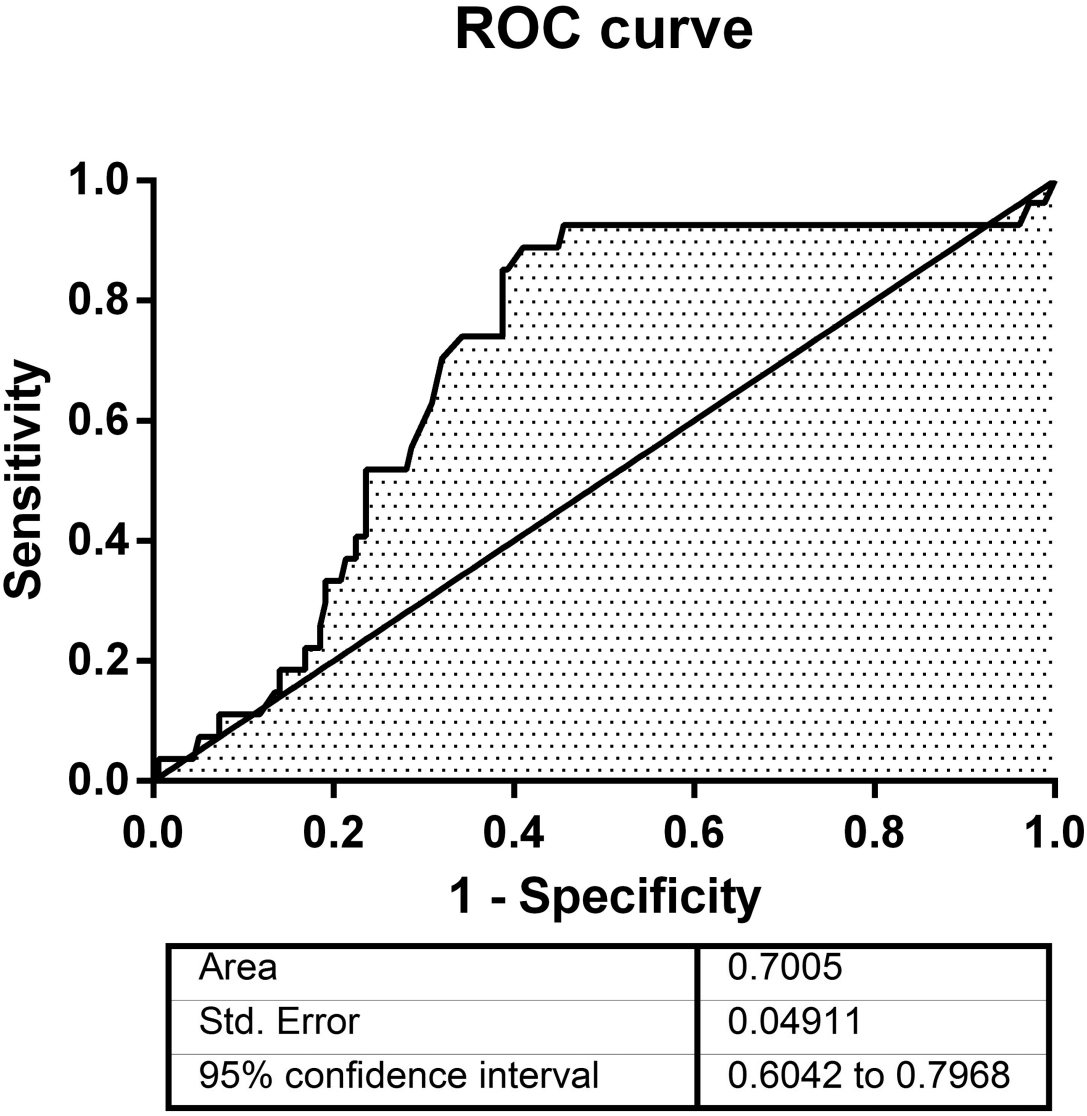
ROC curve of lesion volume.

To evaluate the diagnostic accuracy of LV and compare the predetermined 0.5 mL LV cutoff with our best cutoff value, the corresponding AUC, created by relating LV of clinically sPCa versus benign/iPCa lesions, was assessed (Figure 4).

Our results confirmed the diagnostic significance of the predetermined 0.5 mL cutoff value (Figure 4). The AUC was 0.7 (CI 0.6-0.79) and the best cutoff value we measured was 0.495 mL, corresponding to 85.2% sensitivity and 61.2% specificity.

The diagnostic accuracy of LVencod (LV categorized in two levels, 0 for LV < 0.5 mL and 1 for LV ≥ 0.5 mL, according to 0.5 mL LV cutoff) was also performed along with those of age, PSA, PV, and PSAD. The resulting AUCs are reported in Table 4. LVencod was the most accurate predictor (AUC = 0.732) (Table 4).

### Bivariate and Multivariate Logistic Regression Analysis Using LV, LVencod, PV, PSA, and PDAD as Predictors of sPCa

Results of bivariate and multivariate analysis, assessed to test the ability of all predictors in determining the presence of sPCa at biopsy, were also reported in Table 4.

In bivariate logistic regression models, LV (*P* = .048) and LVencod (*P* < .001) were significantly associated with the presence of sPCa at biopsy (Table 4). On the contrary, age (*P* = .117), PV (*P* = .051), PSA (*P* = .18), and PSAD (*P* = .294) were not significantly associated with the presence of sPCa (Table 4). In the multivariate logistic regression model, testing the predictors of sPCa at biopsy, LVencod (*P* < .001), PV (*P* = .001), and age (*P* = .007) achieved independent predictor status whereas PSA did not (*P* = .738) (Table 4). In agreement with previous data,^11,12^ the OR of PV indicated a negative association of this variable with sPCa.

Comparing bivariate to multivariate results, in the multivariate model, LVencod showed a higher relationship with sPCA (OR = 26.75) and the model was more accurate (AUC = 0.865) if compared to single sPCa predictors (Table 4).

## Discussion

MpMRI represents the reference standard imaging modality in the detection, staging, treatment monitoring and outcome prediction for PCa. PI-RADS v2.1^1^ classifies the suspicious lesions into 5 categories based on the risk of having sPCa. A consensus was reached regarding the need not to perform the biopsy for score 1 and 2 lesions (clinically significant cancer is highly unlikely or unlikely to be present, respectively) and to perform biopsy for score 4 and 5 lesions (clinically significant cancer is likely or highly likely to be present, respectively). Conversely, there is still not a consensus about how to manage patients with PI-RADS score 3 lesions in which the presence of clinically significant cancer is equivocal. The uncertainty regarding the management of PI-RADS v2.1 score 3 lesion represents one of the most important ambiguities and limitations of this system.^2^ The question is, How should PI-RADS score 3 lesions be managed?

It should be emphasized that several factors beyond the MRI appearance may affect a patient’s clinical management. These include, but are not limited to the number of previous biopsies, diagnosis at the previous biopsy (i.e., ASAP, HGPIN, BPH), age, family history, PSA level, PSA velocity, and other biomarkes).^11-15^ All these factors are usually considered before giving the indication to perform a mpMRI examination. In other words, the indication to perform a fusion-targeted biopsy usually includes both full patient history and mpMRI findings.

As far as the purely radiological aspect is concerned, and in respect of the above clinical parameters, different authors have tried to solve this dilemma. Liddell H et al^16^ in a previous paper concluded that prostate lesions characterized as PI-RADS score 3 are associated with a low likelihood of sPCa presence and that these lesions should not be sampled but only monitored. In contrast, Thompson et al^4^ reported a 26% overall detection rate of PCa among their PI-RADS score 3 lesions series. Among these, 38% were moderate- or high-risk lesions. Recently Scialpi et al.^17^ using a simplified PI-RADS score (S-PI-RADS) by biparametric MRI,^18^ discussed the implication of score 3 lesion management demonstrating that the 3a and the 3b lesions were sPCa in 2.8% and 27.5%, respectively, and suggested the relevance of categorization and management for each lesion.^19^ Besides, Ploussard et al^20^ emphasized the usefulness of pre-treatment diagnostic tools capable of distinguishing iPCa from sPCa and indicated that they should be one of the main goals of urologists in the following years in order to reduce the risk of over diagnosis and overtreatment of iPCa.

Although the use of biochemical markers has recently been suggested as clinical discriminators to indicate which PI-RADS 3 lesions are worthy of biopsy,^21^ to date, there is still no universally accepted discriminators.

At present, the most used criteria to define iPCa are based on the pathologic assessment of the radical prostatectomy specimen. They include three well-established prognostic factors, as described by Ohori et al^[Bibr b7-tju-48-4-268] 22^ and Epstein et al.^7 [Bibr b22-tju-48-4-268]^ (1) a Gleason score ≤6 without Gleason pattern 4 or 5, (2) organ-confined disease (no extra prostatic extension, seminal vesicle invasion, or lymph node involvement); and (3) a tumor volume <0.5 mL. The categorization of the tumor was based on the mass with the largest tumor volume (i.e., the dominant or index tumor).^23^

Even if the measurement of LV with MRI is not still universally accepted as truthful,^24^ several studies have accurately evaluated the correspondence of mpMRI LV with histological tumor volume on radical prostatectomy specimens, finding a positive correlation with an underestimation of mpMRI LV ranging from 4.2% to 5.9% (without shrinkage factor).^6,8^

In accordance with Epstein’s criteria, the present data strongly indicate that the LV classification approach, based on 0.5 mL cutoff, may represent an effective pre-treatment tool to easily discriminate sPCa from iPCa. The AUC (0.732) of LVencod indicated a good diagnostic accuracy in identifying sPCa at biopsy. The choice of performing FTBx on PI-RADS 3b lesions only would avoid 55.1% (113/205 lesions) of unnecessary biopsies and would result in a loss of 1.95% (4/205) sPCa. This percentage is acceptable and much lower if compared to that of the entire PI-RADS V2.1 score 3 category. PI-RADS v2.1 score 3a lesions (LV < 0.5 mL) would be then worthy of accurate clinical and radiological follow-up (e.g., PSA every 6 months with a repeated mpMRI after 1 year) and the evidence that iPCa remains stable over time following the diagnosis^25^ strongly supports this perspective. Such patients would be candidates for biopsy in case of score switches from 3a to 3b category, allowing both early diagnosis and surgical treatment with curative intent.

Our data also demonstrate that the predictive ability of LVencod increased if it was included, along with age and PV, in a multivariate model. In this case, the resulting AUC (0.865) was superior than those of single variables, thus indicating that studies in this direction should be performed with larger populations in order to achieve a risk calculator.

In conclusion, we suggest that the above approach, used alone or in combination with other risk factors in a multivariate model, may represent a simple, easily reproducible, and effective way to solve the ambiguities related to the management of the “gray zone” of PI-RADS v2.1 score 3 lesions. In addition, it may allow to the reduction of nearly 50% of unnecessary biopsies with consequent and, possibly, high decrease of the costs. Also, the above approach, treating PIRADS 3a lesions with a “watchful waiting” way and verifying the evolution of the suspicious lesions, over time, through repeated mpMRI alone, would avoid the adoption of Active Surveillance conduct (periodic biopsy check) for these patients, who are almost totally diagnosed as benign/iPCa (96.5%). Bioptic check, however, would remain strongly recommended when the suspected lesions change their characteristics (lesion volume >0.5 mL and/or PI-RADS score upgrading).

The present study has some limitations. These include its monocentric and retrospective design, first, and, second, the criteria used to define the clinically sPCa which refer to radiological measurements of tumor volume whose role has still to be definitively confirmed. Nevertheless, a reliable correlation between tumor volume and lesion volume had already been demonstrated in different papers and in our previous study,^6^ which comprises some patients considered in the present one. Moreover, the dimensional criterion (evaluated by TC or MRI) is commonly adopted for clinical management of other solid tumors (e.g., in lung, liver, and kidney), and, therefore, we believe that it can be employed also in the PCa model. Finally, all the PI-RADS score 3 lesions were defined using PI-RADS v2.1 system by two very experienced radiologists, therefore, it cannot be excluded that the evaluations may have been subjective (i.e., some cases which were included in our casuistic may have been underestimated to PI-RADS score 2 or up estimated to score 4 by other radiologists).

In conclusion, the present study suggests a useful solution on how to solve the limits related to PI-RADS v2.1 score 3 lesion management encouraging radiologists to adopt mpMRI LV evaluation, used singularly or in combination with patient’s age and PV. The categorization into subcategories 3a and 3b allows for a more accurate management of the score 3 lesions, avoiding biopsy indication for patients who would be almost totally diagnosed as benign/iPCa.

## Figures and Tables

**Table 1.** Summary of Findings on PI-RADS 3 Category and PI-RADS 3a and 3b Subcategories

			**n**			**%**		
**PI-RADs 3 category **(all lesions)			205			100		
**3a subcategory **(lesions <0.5 mL)			113			55.1		
**3b subcategory **(lesions ≥0.5 mL)			92			44.9		
		**Type of Lesion**	**n**	**n in PZ**	**n in TZ**	**% of total**	**% in 3a**	**% in 3b**
PI-RADS 3 (n = 205)	3a (n = 113)	benign (n = 80) **iPCa (n =29 GS 6)**	109	40	69	53.2	96.5	–
	sPCa (GS ≥7)	4	4	0	1.95	3.5	–
	3b (n = 92)	**benign**	69	30	39	33.65	–	75
sPCa (n = 21 GS 6; n = 2 GS 7)	23	14	9	11.2	–	25

N, number of subjects; PZ, proximal zone; TZ, transitional zone; iPCa, indolent prostate cancer; sPCa, significant prostate cancer.

**Table 2.** Variables under Investigation in PI-RADS 3 Category and Subcategories

	**PI-RADs 3 Category**		**3a Subcategory**		**3b Subcategory**		**Comparison 3a vs. 3b**
	Median	IQR	Median	IQR	Median	IQR	*P*
**Age (years)**	65	59-72	65	60-73	63	59-69	.09
**LV (mL)**	0.44	0.21- 0.71	0.23	0.15-0.325	0.745	0.57-1.2	^*^<.001
**PV (mL)**	54	41-83	49	38-67	65.5	41.75-87	^*^.011
**PSA (ng/mL)**	8.1	6-10.7	8.55	6-11.3	8.1	6.2-10	.7
**PSAD (ng/mL/cm** **3** **)**	0.14	0.09-0.21	0.16	0.11-0.23	0.125	0.08-0.21	^*^.033

IQR, interquartile range; LV, lesion volume; PV, prostate volume; PSAD, PSA density.

The comparison of data between PI-RADs 3a and PI-RADs 3b subcategories was performed using the non-parametric Mann–Whitney U-test. ^*^Statistically significant difference.

**Table 3.** Variables under Investigation in the Groups of Benign/Indolent and Significant PCa Lesions

	**Benign/Indolent PCa Lesions**		**Significant PCa Lesions**		
	Median	IQR	Median	IQR	*P*
**Age** **(years)**	64	59-71	68	62.2-73.2	.13
**LV** **(cm** **3** **)**	0.37	0.2-0.67	0.69	0.52-0.83	^*^< .001
**PV** **(cm** **3** **)**	54	42.7-83	38.4	26-64.25	^*^.013
**PSA** **(ng/mL)**	8.4	6-11	7.650	6.65-8.67	.32
**PSAD** **(ng/mL/cm** **3** **)**	0.14	0.09-0.2	0.195	0.095-0.26	.24

LV, lesion volume; PV, prostate volume; PSAD, PSA density.

The comparison of data between the groups of benign/indolent and significant PCa lesions was performed using the non-parametric Mann–Whitney U-test. *Statistically significant difference.

**Table 4.** ROC curves of individual predictor variables in bivariate and multivariate analysis.

	**ROC Curve **	**Bivariate Analysis**	**Multivariate Analysis**
	AUC (95% CI)	OR (95% CI); *P*	OR (95% CI); *P*
**Age**	0.609 (0.471-0.734)	1.051 (.988-1.119); .117	1.112 (1.03-1.2); .007
**LV** **a**	0.7 (0.6-0.792)	1.78 (1-3.15); .048	–
**LVencod**	0.755 (0.638-0.850)	12.94 (3.7-44.8); <.001	26.75 (5.98-119.6); <.001
**PV**	0.326 (0.179-0.473)	0.979 (0.959-1.0); .051	0.96 (0.936-0.935); .001
**PSA**	0.425 (0.316-0.533)	0.917 (0.808-1.04); .18	0.98 (0.848-1.132); .783
**PSAD** **b**	0.581 (0.430-0.732)	7.2 (0.179-296.3); .294	–
**Multivariate** **model** (LVencod, age, PV)	**ROC curve**		
	AUC (95% CI)		
0.865 (0.763-0.968)		

LV, lesion volume; LV encod, lesion volume encoded; PV, prostate volume; PSAD, PSA density; AUC, area under the curve; CI, confidence interval; OR, odds ratio.

^a^Not included in the multivariate model because of the strong correlation with LVencod; ^b^not included in the multivariate model, *P* ≥ .25.
